# Case Report: Chronic eosinophilic pneumonia with acute-like presentation and diagnostic challenges

**DOI:** 10.3389/fmed.2026.1841565

**Published:** 2026-06-03

**Authors:** Alexandra-Simona Zamfir, Adina Simona Mihailovici, Florin Mihai, Carmen Lăcrămioara Zamfir, Gabriela Bordeianu, Bogdan-Mihnea Ciuntu, Marcela Brînză, Tudor Andrei Cernomaz

**Affiliations:** 1 Department of Medical Sciences III, Pulmonology, Faculty of Medicine, “Grigore T. Popa” University of Medicine and Pharmacy, Iasi, Romania; 2 Clinical Hospital of Pulmonary Diseases Iași, Iasi, Romania; 3 Department of Surgery II, Radiology, Faculty of Medicine, “Grigore T. Popa” University of Medicine and Pharmacy, Iasi, Romania; 4 Department of Morpho-Functional Sciences I, Faculty of Medicine, University of Medicine and Pharmacy “Grigore T. Popa”, Iasi, Romania; 5 Department of Morpho-Functional Sciences II, University of Medicine and Pharmacy “Grigore T. Popa”, Iasi, Romania; 6 Department of Surgery I, Faculty of Medicine, “Grigore T. Popa” University of Medicine and Pharmacy, Iasi, Romania

**Keywords:** acute-like presentation, airflow limitation, bronchoalveolar lavage, chronic eosinophilic pneumonia, corticosteroid therapy, diagnostic challenge, relapse

## Abstract

**Background:**

Chronic eosinophilic pneumonia (CEP) is a rare eosinophilic lung disease characterized by pulmonary and peripheral blood eosinophilia. It predominantly affects middle-aged women and remains a diagnosis of exclusion based on compatible clinical and imaging findings together with the absence of secondary causes. Distinguishing CEP from acute eosinophilic pneumonia (AEP) or infectious processes may be challenging, particularly in atypical presentations. Systemic corticosteroids represent the standard therapy and typically produce rapid clinical improvement, although relapse is common after treatment tapering or withdrawal.

**Case presentation:**

We report the case of a 65-year-old female smoker presenting with persistent cough, dyspnea, and chest pain, initially treated as community-acquired pneumonia. The diagnostic work-up included laboratory testing, pulmonary function assessment, high-resolution computed tomography (HRCT), bronchoscopy with bronchoalveolar lavage (BAL) and bronchial biopsy. Peripheral eosinophilia and elevated inflammatory markers were observed. HRCT demonstrated bilateral upper-lobe-predominant consolidations associated with centrilobular opacities and tree-in-bud changes. Microbiological and parasitological investigations were negative. BAL did not demonstrate eosinophil predominance; however, bronchial biopsy revealed eosinophil-rich inflammatory infiltrates. After exclusion of infectious and parasitic causes and integration of clinical, imaging, and histopathological findings, a diagnosis of chronic eosinophilic pneumonia was established. Oral methylprednisolone (16 mg/day) was administered for approximately 8–10 weeks, followed by gradual tapering to 8 mg/day, resulting in marked clinical and radiological improvement. One month after corticosteroid withdrawal, symptoms recurred, requiring treatment reintroduction. Follow-up HRCT demonstrated complete resolution of the consolidations. An exploratory attenuation-based aeration analysis suggested regional hyperaeration in areas previously affected by consolidation, although its clinical significance remains uncertain.

**Conclusion:**

This case illustrates the diagnostic complexity of CEP when acute-like clinical features, atypical imaging findings, and discordant bronchoalveolar lavage results coexist and highlights the importance of integrating clinical, imaging, microbiologic and histopathological data to establish the diagnosis.

## 1 Introduction

Chronic eosinophilic pneumonia (CEP), first described in the late 1960s, is a rare interstitial lung disease characterized by eosinophilic infiltration of both peripheral blood and pulmonary tissue, as demonstrated by bronchoalveolar lavage (BAL) or histopathologic examination. CEP commonly affects middle-aged women and typically presents with a subacute course, including cough, dyspnea, and constitutional symptoms, often accompanied by peripheral eosinophilia (1–3). High-resolution computed tomography (HRCT) classically demonstrates peripheral or subpleural consolidations and ground-glass opacities, although imaging patterns may vary (3, 4).

Despite these characteristic features, CEP remains a diagnosis of exclusion and may pose significant diagnostic challenges, particularly in cases with atypical clinical evolution, non-specific functional findings, or unusual imaging patterns. Differentiation from other eosinophilic lung diseases—including allergic bronchopulmonary aspergillosis, eosinophilic granulomatosis with polyangiitis, parasitic infections, and acute eosinophilic pneumonia—can be particularly difficult when presentation deviates from the classical subacute phenotype (5, 6).

Although its reported incidence is considered to be below 3%, the true prevalence of the disease remains uncertain, largely due to underdiagnosis and its often non-specific clinical manifestations, such as cough or dyspnea (3). Eosinophilic pneumonias are characterized by an increased accumulation of eosinophils within the lung parenchyma, typically identified through bronchoalveolar lavage or tissue biopsy. They are classified according to the onset and course of symptoms as either acute, more commonly affecting men, or chronic, which predominantly occurs in women. The acute form often presents with severe manifestations, potentially progressing to hypoxemia and respiratory failure, whereas the chronic form generally exhibits nonspecific clinical features, combining systemic and respiratory symptoms resembling those of pneumonia. The diagnosis of chronic eosinophilic pneumonia is based on somewhat loose criteria which include: symptoms lasting more than 2 weeks, suggestive imagistic data, presence of eosinophils in bronchoalveolar lavage or tissues and exclusion of alternative causes (1). Systemic corticosteroids remain the mainstay of treatment and usually produce rapid clinical improvement, although relapse is common after dose reduction or withdrawal (1).

We report a diagnostically challenging case of CEP in a 65-year-old smoker, initially misinterpreted as community-acquired pneumonia due to its acute-like presentation. The main distinctive feature of this case is the coexistence of discordant diagnostic elements, including a neutrophil-predominant bronchoalveolar lavage, atypical imaging findings such as tree-in-bud opacities, and histopathologic confirmation of eosinophilic inflammation. These findings highlight the limitations of relying on single diagnostic modalities and emphasize the importance of an integrated clinicopathologic approach. Additionally, an exploratory CT-based aeration analysis is presented as a hypothesis-generating observation. This case illustrates that CEP may present with misleading clinical, imaging, and bronchoalveolar findings, potentially delaying diagnosis, and emphasizes the importance of maintaining diagnostic suspicion even in the absence of typical features.

## 2 Case report

### 2.1 Initial presentation and empirical management

A 65-year-old woman with a 30-pack-year smoking history presented with a two-week history of productive cough, progressive dyspnea, and anterior chest pain, initially managed as community-acquired pneumonia, but with an atypical clinical and radiological course. She lived in a rural area and reported no clear occupational exposures. Her medical history included arterial hypertension, type 2 diabetes mellitus, and gastric ulcer, with no prior history of asthma or eosinophilic disease. She had a body mass index (BMI) of 30.5 kg/m2. There was no relevant family history. Symptoms had developed subacutely over approximately 14 days prior to admission (Day 0), with progressive worsening.

At hospital admission (Day 14), the patient was afebrile, with a respiratory rate of 20 breaths per minute, blood pressure of 140/90 mmHg, and mild hypoxemia (SpO_2_ 90%). Respiratory examination revealed bilateral basal crackles and diffuse rhonchi. Cardiac auscultation and abdominal examination were unremarkable. There was no palpable lymphadenopathy or peripheral edema, and the remainder of the physical examination was within normal limits.

Laboratory evaluation demonstrated an inflammatory syndrome, with elevated C-reactive protein (46.5 mg/L) and erythrocyte sedimentation rate (60 mm/h), associated with peripheral eosinophilia (absolute eosinophil count 1.55 × 10⁹/L; reference range 0.04–0.54 × 10^9^/L). Routine microbiological investigations were negative, including sputum culture (nonspecific flora), acid-fast bacilli testing, SARS-CoV-2 PCR, and respiratory viral panel.

**Figure 1:**
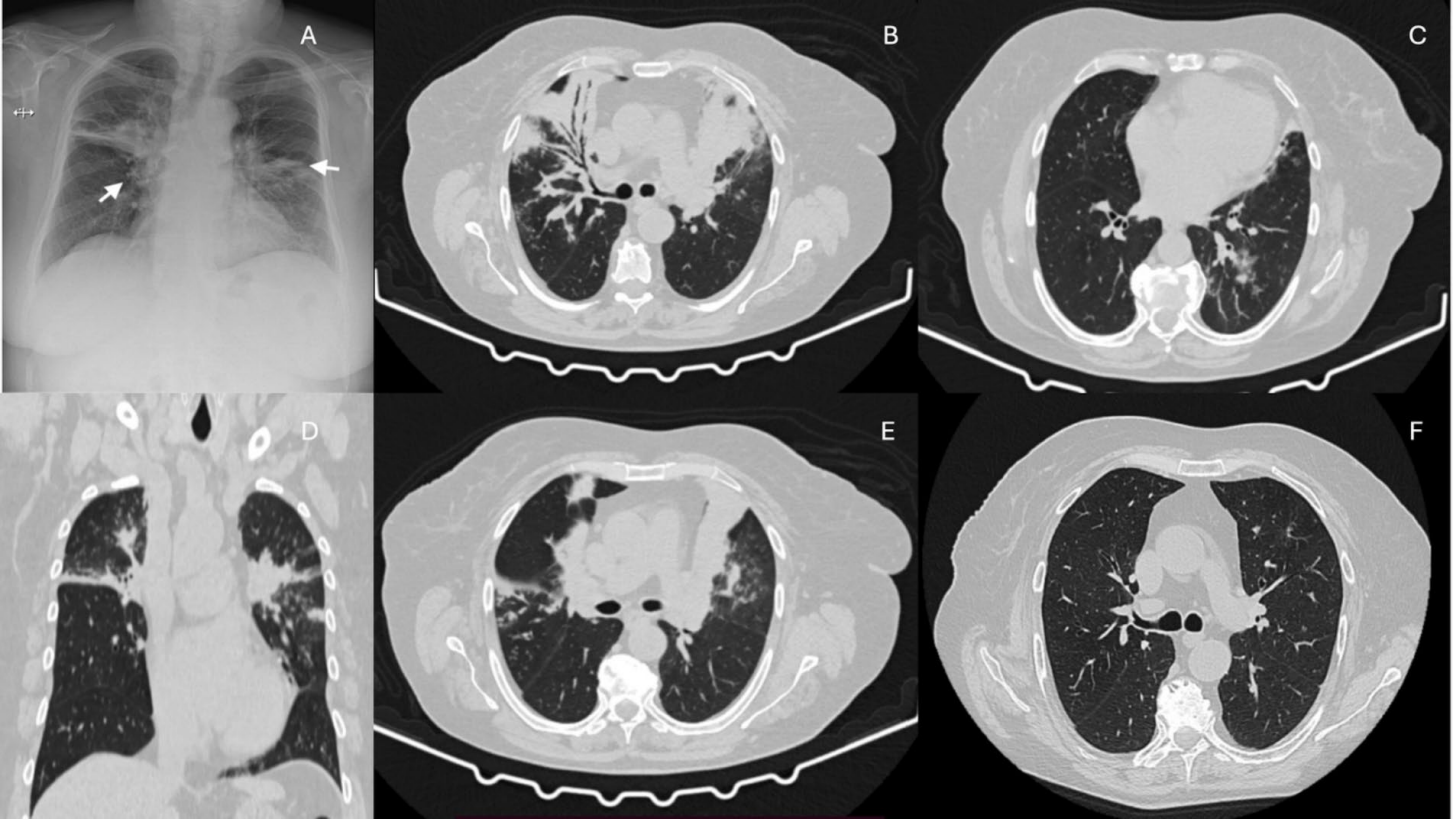
Imaging findings at presentation and follow-up. **(A)** Initial chest radiograph (postero–anterior view) showing bilateral perihilar opacities (arrows) with partial confluence and trans-fissural extension (Day 14). **(B)** Axial HRCT (lung window) demonstrating centrilobular ground-glass and denser opacities, as well as branched opacities with a “tree-in-bud” appearance. **(C)** Axial HRCT (lung window) showing centrilobular branching nodules with tree-in-bud pattern in the middle lobe, together with left-sided consolidation. **(D)** Coronal HRCT reconstruction (lung window) illustrating consolidation of the anterior segment, right upper-lobe with well-defined air bronchogram pattern. **(E)** Axial HRCT (lung window) demonstrating bilateral centrilobular and branched micronodules in the lower lobes. **(F)** Follow up axial HRCT (lung window) showing complete resolution of the upper lobes consolidation (Day 150).

Initial postero-anterior chest radiography demonstrated bilateral perihilar opacities of moderate density with a tendency toward confluence, irregular margins, and trans-fissural extension, associated with thickening of the right major fissure and partial relaxation of the right hemidiaphragm (Figure 1A).

In this clinical context, a working diagnosis of community-acquired pneumonia (risk class III) was established, and empiric treatment with intravenous ceftriaxone (3 g/day), bronchodilators, mucolytics, and systemic corticosteroids was initiated. Smoking-related obstructive airway disease was also considered in the differential diagnosis, given the patient’s history and clinical findings.

Despite initial therapy, respiratory symptoms persisted over the following days (Day 14–18), with only partial clinical improvement. Pulmonary function testing performed on Day 18 demonstrated a moderate obstructive ventilatory defect with negative reversibility (FVC 2.22 L, 80%, z score −1.25; FEV_1_ 1.64 L, 71%, z score −1.80; FEV_1_/FVC 73%, z score −0.57), associated with mildly reduced DLCO and a supranormal transfer coefficient (DLCO—17.01 mL/min/mmHg, 75%, z score −1.63; DLCO corrected—18 mL/min/mmHg, 79%, z score—1.35; transfer coefficient—5.26 mL/min/mmHg, 119%, z score 0.56), supporting airflow limitation without establishing a specific diagnosis. Inhaled budesonide/formoterol (320/9 mcg) therapy was therefore initiated. At this stage, the persistence of symptoms despite appropriate antibiotic therapy, together with peripheral eosinophilia, raised suspicion for a non-infectious etiology.

### 2.2 Diagnostic reassessment

Chest CT performed on Day 19 revealed consolidation in the anterior segment of the right upper-lobe with well-defined air bronchogram pattern, associated with centrilobular ground-glass and denser opacities, as well as branched opacities with a “tree-in-bud” appearance (Figure 1B). In the left upper-lobe, there was extensive consolidation in the anterior segment and multiple smaller confluent foci of consolidation within the lingula; centrilobular ground-glass and denser opacities and “tree-in-bud” patterns were also noted (Figures 1C,D). Because tree-in-bud opacities are atypical for classic CEP and often raise concern for infection or small-airways disease, this imaging pattern initially complicated the diagnostic interpretation. Additionally, clusters of centrilobular and branched micronodules were identified along the peribronchovascular structures in the lateral and posterior segments of the lower lobes (Figure 1E). Bilateral mediastinohilar lymphadenopathy was also noted, involving the right retrotracheal, superior paratracheal, subcarinal, paraesophageal, hilar, and interlobar stations, as well as the corresponding left paratracheal, hilar, and interlobar regions.

These findings broadened the differential diagnosis beyond simple bacterial pneumonia and prompted further investigations. At this stage, the diagnostic process was challenging due to the coexistence of acute-like clinical features and atypical imaging findings, including tree-in-bud opacities, which raised concern for infectious or small-airway disease.

Bronchoscopy was performed on Day 20, revealing a normal right bronchial tree up to the segmental level. In the left main bronchus, an adherent whitish deposit was observed on edematous, infiltrated mucosa, and biopsies were obtained. Abundant organized secretions and bronchial casts were aspirated, particularly from the upper-lobes. Samples were collected for bacteriological, mycological and acid-fast bacilli (AFB) testing, including PCR analysis. Bronchoalveolar lavage (BAL) was subsequently performed, showing saprophytic flora without pathogenic significance and a neutrophil-predominant inflammatory pattern rather than a clearly eosinophil-predominant differential. Differential cell count showed neutrophils as the dominant population, with only a minor eosinophilic component (exact percentages unavailable due to sample processing limitations). Histopathologic examination of the bronchial biopsy demonstrated a moderately dense inflammatory infiltrate in the lamina propria, composed of lymphocytes, numerous plasma cells and frequent eosinophils, with epithelial exocytosis of lymphocytes, neutrophils and eosinophils. No evidence of invasive tumor was observed on serial sections or immunohistochemical analysis. The diagnostic process remained challenging, further complicated by the neutrophil-predominant BAL findings in the context of suspected eosinophilic lung disease.

### 2.3 Final diagnosis and treatment

After exclusion of common parasitic causes (Days 21–23) through stool examination and serologic testing for *Toxocara* and *Echinococcus*, the final diagnosis of chronic eosinophilic pneumonia (CEP) was established by integrating the clinical, imaging, microbiological, and histopathological findings, despite several atypical features, including the relatively acute onset, neutrophil-predominant BAL, and tree-in-bud pattern on imaging.

Oral methylprednisolone (16 mg/day) was initiated on Day 24 and continued for approximately 8 weeks, followed by gradual tapering to 8 mg/day based on clinical and radiological improvement. No treatment-related adverse events were observed. However, approximately 1 month after treatment withdrawal (~Days 110–120), the patient experienced symptom recurrence with eosinophilia, requiring reintroduction of methylprednisolone (16 mg/day), again with favorable response. Anti IL-5 therapy was also considered, but delayed due to local regulations for compensation (requiring multiple corticosteroid rounds). Blood glucose monitoring and gastrointestinal prophylaxis with proton pump inhibitors were performed during corticosteroid therapy.

### 2.4 Follow-up

At follow-up (~Day 150), chest CT demonstrated complete resolution of the consolidations, while mediastinal lymph nodes remained largely unchanged. This examination was performed approximately 3 months after the initial CT and 2 weeks after reintroduction of corticosteroid therapy (Figure 1F).

Treatment was well tolerated, and no adverse effects related to corticosteroid therapy were reported during follow-up. Adherence to therapy was assessed through clinical follow-up and patient report, with good compliance throughout the treatment period. The patient declared significant symptomatic improvement under corticosteroid therapy, with recurrence of dyspnea and cough after treatment withdrawal.

**Figure 2:**
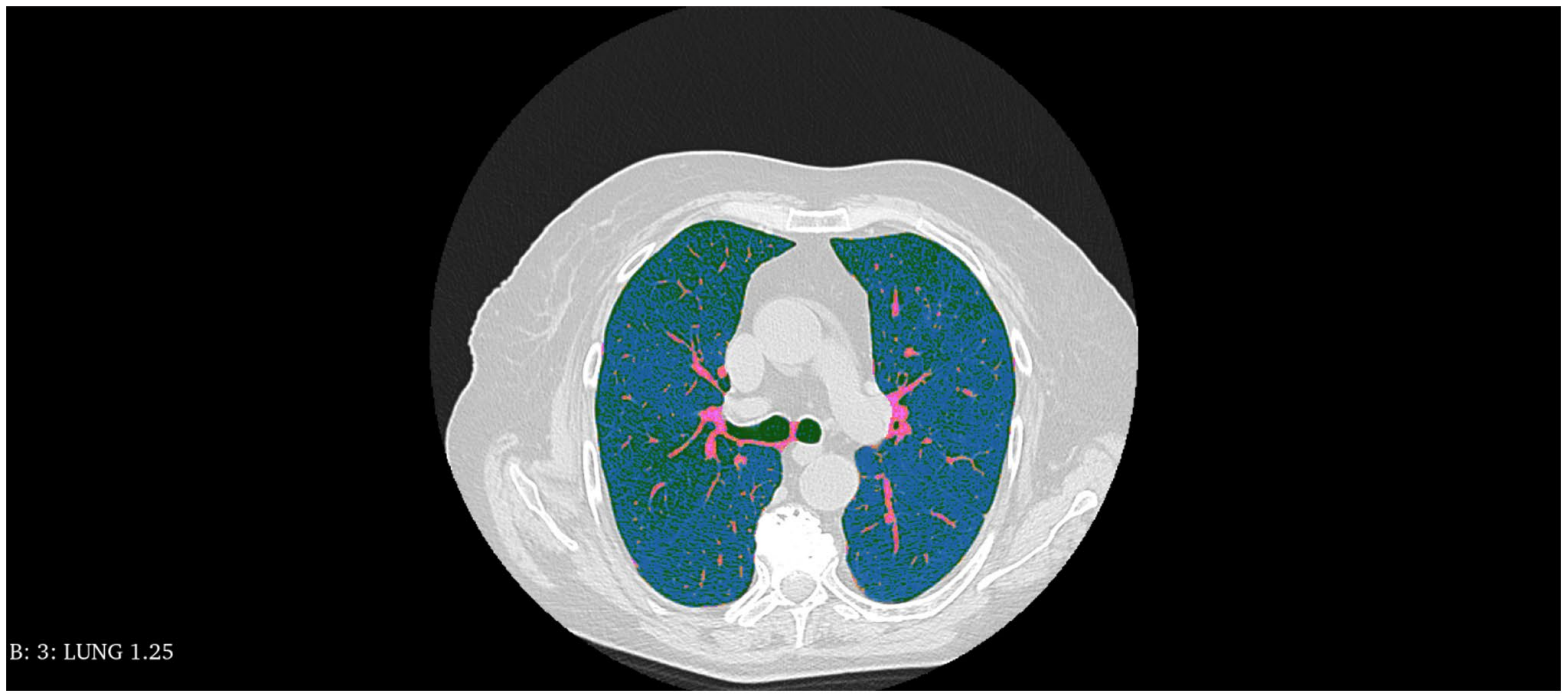
Exploratory attenuation-based aeration mapping on follow-up inspiratory CT scan. Segmentation was performed using 3D Slicer (version 5.8.1) based on predefined attenuation thresholds. Hyperinflated regions (−1,000 to −901 HU; shown in dark green) are identified in the anterior segment of the right upper-lobe, the middle lobe, and the left upper-lobe (culmen). Normally aerated lung regions (−900 to −501 HU; shown in blue) largely correspond to areas that showed consolidation on CT performed 3 months earlier.

Despite radiologic improvement on follow-up chest CT, pulmonary function testing, performed shortly thereafter, did not show significant recovery of airflow limitation or gas transfer. Because of the persistence of functional abnormalities, an exploratory *post hoc* aeration assessment was subsequently performed (Day 155) on the follow-up inspiratory CT using 3D Slicer version 5.8.1, with the following attenuation thresholds: hyper-aerated volume (−1,000 to −901 HU), normally aerated volume (−900 to −501 HU), poorly aerated volume (−500 to −101 HU), and non-aerated volume (−100 to +100 HU) (7, 8). This analysis was descriptive in nature and had not been prospectively planned as a validated quantitative imaging endpoint. The exploratory segmentation revealed areas of hyperinflation that largely overlapped with the regions of consolidation observed 3 months earlier (Figure 2).

This finding is challenging to interpret. Possible explanations include incomplete resolution of inflammation leading to small-airway obstruction and dynamic hyperinflation, or a residual consequence of prior alveolitis. Alternatively, the hyperinflation may have been preexisting as the patient had risk factors for COPD; should this be the case, a causal relationship might be suspected—either hyperinflation or small-airway disease may favor eosinophils accumulation. Since no previous CT images were available, this observation was interpreted as hypothesis-generating only and not as evidence of a causal relationship between CEP and persistent hyperinflation. The patient’s clinical course is summarized in Table 1. This case report was prepared in accordance with the CARE guidelines.

**Table 1:** Timeline of key clinical events, diagnostic assessments, and therapeutic interventions during the patient’s clinical course.

Time	Clinical course	Key findings	Management and outcome
Day 0–14	Progressive onset of productive cough, dyspnea, and chest pain	–	Symptom progression
Day 14 (Admission)	Hypoxemia (SpO_2_ 90%), bilateral crackles	Inflammatory syndrome, eosinophilia; chest X-ray: bilateral perihilar opacities	Empiric antibiotics +bronchodilators+ corticosteroids→partial response
Day 14–18	Persistent respiratory symptoms	–	Incomplete clinical improvement
Day 18	–	Pulmonary function tests: obstructive pattern, reduced DLCO	Inhaled budesonide/formoterol initiated
Day 19	–	Chest CT: bilateral consolidations, ground-glass opacities, tree-in-bud pattern, lymphadenopathy	Raised suspicion for non-infectious etiology
Day 20	Bronchial inflammation, secretions	Bronchoscopy + BAL (neutrophil-predominant), biopsy: eosinophilic infiltrate	Infection excluded
Day 21–23	–	Parasitic testing negative	Diagnosis refined
Day 24	Persistent symptoms	Integrated clinicopathologic findings	Diagnosis of chronic eosinophilic pneumonia (CEP); oral corticosteroids initiated
Day 24–80	Progressive clinical improvement	–	Corticosteroid therapy with tapering → marked improvement
Day 110–120	Recurrence of symptoms	Eosinophilia relapse	Corticosteroids reintroduced → rapid response
~Day 150	Follow-up evaluation	CT: complete resolution of consolidations	Radiologic resolution
Day 150–155	Persistent functional limitation	PFT: persistent obstruction; CT aeration: regional hyperinflation	Residual functional impairment
>Day 150	Stable under treatment	–	Maintenance therapy (low-dose corticosteroids + indacaterol)

## 3 Discussion

Eosinophilic lung diseases (ELD) encompass a large variety of disorders primarily characterized by the abnormal accumulation of eosinophils within pulmonary tissues and sometimes in peripheral blood. They include idiopathic—such as eosinophilic pneumonias and hypereosinophilic syndromes (lymphocytic or myeloproliferative forms)—or secondary eosinophilic lung diseases as a result from a variety of conditions. These include allergic bronchopulmonary aspergillosis (ABPA), eosinophilic granulomatosis with polyangiitis, drug-induced reactions, Löffler’s syndrome, parasitic infections (including *Ascaris*, *Strongyloides*, *Paragonimus* or *Toxocara*), and, in rare cases, pathogens such as SARS-CoV-2 or influenza virus (9–12).

Among these, CEP typically follows a subacute course and is characterized by peripheral eosinophilia, suggestive imaging findings, and a favorable response to corticosteroid therapy. However, as illustrated by the present case, the diagnosis may become challenging when clinical, imaging, and bronchoalveolar findings deviate from the classical description.

Although CEP is classically described in younger or middle-aged women, our patient was older and had no prior history of asthma or other eosinophilic disease (13). Her significant smoking history and obstructive ventilatory pattern introduced additional diagnostic complexity, as chronic obstructive airway disease could also account for part of the persistent functional impairment.

In our patient, the initial presentation closely mimicked community-acquired pneumonia, with acute-like symptom onset, inflammatory syndrome, and bilateral pulmonary opacities. In addition, smoking history, wheezing, and the presence of airflow limitation supported consideration of smoking-related obstructive airway disease.

What made this case particularly challenging was the coexistence of several elements that were not typical for classical CEP and complicated the diagnostic process. The relatively short duration of symptoms, elevated inflammatory markers, and the presence of tree-in-bud opacities broadened the differential diagnosis and raised suspicion for infectious or small-airway disease. Furthermore, the neutrophil-predominant pattern observed on BAL was discordant with the expected eosinophilic profile, further delaying diagnostic clarification. These findings should be interpreted with caution. Prior corticosteroid exposure is known to reduce eosinophil counts in BAL fluid and may lead to atypical cellular patterns. In addition, sampling variability and disease heterogeneity may contribute to non-representative BAL profiles, particularly in focal or patchy disease.

Various differential diagnoses were considered and rejected as diagnosis criteria were not met: allergic bronchopulmonary mycosis (ABPM) (14) various fungal and parasitic infections, cryptogenic organizing pneumonia (15), eosinophilic granulomatosis with polyangiitis (16). Hypereosinophilic syndrome (HES) was also taken into account, but the clear rapid response to steroids strongly pointed to the CEP diagnosis.

The differential diagnosis between acute eosinophilic pneumonia (AEP) and CEP was particularly relevant in this context (Table 2) (1–3, 9). Although some features overlapped with AEP, including the acute-like presentation and inflammatory profile, the absence of severe respiratory failure, the presence of marked peripheral eosinophilia, and the characteristic relapse after corticosteroid withdrawal supported the diagnosis of CEP. It is worth noting that this episode occurred in the summer; as there are reports linking an increased incidence of AEP to this particular season at least for younger patients (17, 18). Rather than representing a strict dichotomy, this case may reflect a continuum within eosinophilic pneumonias, with CEP presenting with acute-like features.

**Table 2:** Comparative features supporting acute eosinophilic pneumonia (AEP) versus chronic eosinophilic pneumonia (CEP), with corresponding findings in the present case.

Feature	AEP-like pattern	CEP-like pattern	Findings in the presented case
Symptom onset	Acute (days–weeks)	Subacute (weeks–months)	Subacute (~14 days)
Clinical severity	Often severe, may progress to respiratory failure	Usually mild–moderate, rarely severe	Mild hypoxemia (SpO2 90%), no respiratory failure
Peripheral eosinophilia	Mild or delayed	Marked, present at diagnosis	Marked (1.55 × 10^9^/L - admission)
Inflammatory markers	Frequently elevated	Variable	Elevated CRP and ESR
Smoking association	Strong (especially recent exposure/change)	No clear association	Significant smoking history
Bronchoalveolar lavage	Mixed cellularity, often neutrophil-predominant early	Eosinophil-predominant	Neutrophil-predominant (atypical)
Imaging (CT)	Diffuse or patchy GGOs, random distribution; possible nodules	Peripheral consolidations, upper lobe predominance	Atypical: consolidations + tree-in-bud pattern
Pleural effusion	Relatively common	Rare	Absent
Response to corticosteroids	Rapid	Rapid	Rapid clinical and radiological improvement
Relapse after treatment	Rare	Common	Present (~Day 110 to 120)
Overall interpretation	AEP	CEP	Overall pattern consistent with CEP despite atypical features

The follow-up CT aeration analysis should be interpreted with caution, although to our knowledge, this type of aeration mapping has not been previously reported in CEP. Even though regional hyperaeration was observed in areas overlapping prior consolidations, this was an exploratory descriptive finding rather than a validated quantitative endpoint. Several major confounders limit interpretation, including the absence of pre-disease CT, the lack of expiratory imaging, the patient’s substantial smoking history, and the possibility of pre-existing air trapping related to small-airways disease or COPD.

We may hypothesize a potential link between obstructive and eosinophilic respiratory disorders—possibly involving IL33; a molecule functioning as an alarmin following an either endothelial/epithelial injury. IL33 will trigger type 2 innate lymphoid cells and macrophages leading to eosinophil recruitment through IL5 and IL13 pathways in the former case and IL18, IL4, IL5 and eotaxin in the latter (19). IL33 driven inflammation might also explain some different features of CEP versus AEP; there is compelling data suggesting IL33 levels are significantly and consistently higher in AEP versus CEP—a small study found significant higher levels of IL33 and IL5 in BAL fluid for AEP patients (20). The presence of higher IL33 in the BAL fluid, but not in the serum of AEP patients, might support the hypothesis of epithelial origin—following an airway injury rather than an endothelial one. Even when considering CEP alone, different molecular mechanisms may define different subgroups; IL33 had higher levels in the CEP relapse subgroup in a retrospective study which included various eosinophilic conditions (21).

IL33 has been also linked to COPD related eosinophilic inflammation of the airway, with elevated concentrations observed in both serum and exhaled respiratory condensate of patients exhibiting sputum eosinophilia (22). This mechanism may represent a plausible explanation for the relationship we observed between eosinophilic pneumonia–related consolidations and the persistence of ventilatory abnormalities.

Several limitations should be acknowledged. The absence of pre-disease imaging precluded assessment of pre-existing lung abnormalities, and the lack of expiratory CT limited evaluation of air trapping. In addition, the aeration analysis was exploratory and not based on a validated quantitative protocol.

Exploring the molecular mechanisms responsible for EP also sheds light on therapy options. Corticosteroids have been used with favorable results, but there is no current consensus on optimal dose, treatment duration and optimal strategy to prevent relapses (3). There is limited, but compelling data—mainly originating from case reports and case series suggesting a role for anti IL5 or anti IL13 agents such as mepolizumab as alternatives to corticosteroids when side effects are significant and there are no economic constraints (23). There is higher quality data published on the efficacy of mepolizumab on hypereosinophilic syndrome—an entity encompassing various forms of idiopathic eosinophilic pneumonias since its definitions includes the presence of an eosinophil count superior to 1,500 elements/mmc and organ damage in the absence of an evident cause (such as parasitic, infectious, neoplastic or allergic) (6). New therapeutic approaches have explored the use of monoclonal antibodies in the management of CEP; however, their efficacy and long-term impact remain insufficiently investigated (11).

## 4 Patient perspective

The patient reported marked improvement in respiratory symptoms during corticosteroid therapy, with significant relief of dyspnea and cough. However, following corticosteroid discontinuation, symptoms recurred, impacting daily activities and overall functional status. Upon reintroduction of corticosteroids, respiratory condition improved again. The treatment was well tolerated, with no significant adverse effects reported, although the patient expressed concerns regarding adherence to a low-salt diet.

## 5 Conclusion

Chronic eosinophilic pneumonia remains a rare and possibly underdiagnosed entity that requires careful differentiation from other eosinophilic lung diseases, particularly when atypical clinical or imaging features are present. Diagnosis is exclusion based and AEP, ABPM, HES and various fungal and parasitic diseases should be considered. This case highlights the diagnostic complexity of chronic eosinophilic pneumonia when discordant clinical, imaging bronchoalveolar, and histopathological findings coexist. Although systemic corticosteroids usually produce rapid clinical improvement, relapses are frequent and long-term functional consequences might persist. The relapsing course observed in this case also reflects the persistent inflammatory nature of eosinophilic lung diseases and highlights the potential need for alternative or steroid-sparing therapeutic approaches in selected patients, such as anti IL5 agents.

## Data Availability

The original contributions presented in the study are included in the article/supplementary material, further inquiries can be directed to the corresponding author.
